# Reliability of Smartphone Accelerometers for Measuring Gait During Data Collection Over Zoom

**DOI:** 10.1089/tmr.2022.0011

**Published:** 2022-06-28

**Authors:** Nancy T. Nguyen, Jefferson W. Streepey

**Affiliations:** Department of Kinesiology, School of Health and Human Sciences, Indiana University—Purdue University Indianapolis, Indianapolis, Indiana, USA.

**Keywords:** biomechanics, reliability, telemedicine, wearable sensors

## Abstract

This study examined whether gait data could be reliably collected by homebound participants using iPhones under online supervision. Eighteen healthy young adults met with investigators through Zoom and installed an app to record acceleration from their iPhones' accelerometers. Half of the subjects walked normally; the other half walked while spelling words backward. During the gait tasks subjects recorded their anterior-posterior (AP), medial-lateral (ML), and vertical (V) accelerations. Data collection was repeated the following week. Seven maximum and minimum peak accelerations in the AP, ML, and vertical directions associated with events in gait were determined. Significant main effects of week and direction were observed for the first and second vertical acceleration measures. Cronbach alpha values were >0.60 for all acceleration measures, but the maximum and minimum AP accelerations that showed fair to good levels of consistency. The findings suggest gait data collected inside the home setting may be of clinical use.

## Introduction

Telemedicine, which enables long-distance medical communication between clinician and patient, has been on the rise for the past 40 years. There are many benefits of telemedicine such as its cost-efficiency, an increased opportunity for patient monitoring, and new avenues for offering patient education.^1,2^ In addition, there is a positive perception of telemedicine among clinicians and patients.^3^ Although popular, a drawback of telemedicine is the difficulty in making accurate assessments when standardized validated technology is not always available to the patients.^4^

In clinical settings, gait is used to measure overall health status. Gait assessments can be used to assess disease progression and predict adverse effects such as falls and injuries due to falls.^5^ Traditionally, the assessment of gait has been limited to laboratory and clinic visits by the patient. Within the past decade however, health care and gait clinics have started to use smartphone devices for simple assessments of gait and balance.^5–7^ The triaxial accelerometers embedded within smartphones have been shown to be capable of assessing body sway and reliably detecting gait parameters such as gait variability and swing duration.^8–13^

In each one of these studies, the type of phone utilized was controlled, and the experiments were conducted in a laboratory setting. To be an effective tool for telemedicine, smartphone-driven gait analysis needs to be reliable when conducted outside of the laboratory or clinic and accommodating to a variety of types of smartphones. The purpose of this study was to determine if gait data could be reliably recorded in the home under the supervision of experimenters through Zoom using iPhones even when the generation of iPhone was not specified.

## Materials and Methods

### Participants

Eighteen healthy young adults ages 18–24 (14 females and 4 males) who were free from injury and without a history of musculoskeletal impairments were asked to participate in the study. The experimental protocol was approved by the Indiana University Institutional Review Board and was performed in accordance with the Declaration of Helsinki. All participants provided verbal consent before data collection.

### Testing apparatus

Subjects met with investigators on Zoom (Zoom Technologies, Inc., San Jose, CA) to conduct the experiment and were directed through the data collection setup and protocol. Participants used their personal iPhones (Apple, Inc., Cupertino, CA) that have a built-in triaxial accelerometer. Any generation of iPhone was included, but all were capable of collecting acceleration data of ±2 g at 50 Hz. Subjects downloaded a data collection app, Vernier graphical analysis GW (Vernier Software and Technology, Beaverton, OR) to collect anterior-posterior (AP), medial-lateral (ML), and vertical (V) acceleration data. During data collection phones were placed in portrait mode and turned upside down (the charger port facing the participants head). Smartphones were held upside down so the direction of the vertical acceleration experienced during foot striking would match the positive ground reaction forces. Participants held their phones steady in this orientation with it positioned near the belly button.

### Procedure

Over Zoom, investigators explained how to set up the walking path. Participants were asked to find a space where they could take 12 steps (∼8 m). Participants started at one end of the room where the Zoom camera was placed, walked 12 steps away from the camera, and placed an item such as a cone or water bottle to mark the end of those steps. They were then instructed to turn around and count the same number of steps back toward the camera before placing another item to mark that spot.

Once the walking course was set, participants were asked to walk this path at a self-selected, comfortable pace starting at the spot closest to the camera, walking down the path, taking a 180° turn at the first marker, and returning to the start position. Trials were initiated by the participants pressing the “collect” button on the data collection app downloaded onto their phones and verbalizing that to the investigators that they had done so. The investigators would time 5 sec and verbalize for the participants to start walking. The trial ended by the participants back at the start position, investigators timing five more seconds and verbalizing that the participant could end the data collection for that trial.

Half of the participants participated in testing in a single task (ST) condition (walking only). To introduce some small variability that may mirror gait in individuals with mild impairments, the other half of the subject population completed a dual task (DT). DT participants were asked to spell words backward while walking their designated path. Words were taken from a random word generator, and words with less than six letters were chosen.

A total of eight trials were done with the first three trials being practice trials and the last five trials being recorded trials. Participants met with investigators again a week later to repeat the study.

### Data analysis

iPhones collected AP, ML, and V acceleration data through the app at 50 Hz. A custom MATLAB (MathWorks, Natick, MA) program was used to low-pass filter the raw data at 12 Hz using a fourth-order Butterworth filter. The program plotted the vertical acceleration to show the third step walking away from the camera and the third step on the return. These steps were determined through visual inspection of plotted vertical acceleration data. Peaks occurring within the ML, AP, and V acceleration data for each step were identified, these points have been shown to correspond with specific gait events; heel strike (minimum ML [MinML], maximum AP [MaxAP], and V1), loading response (maximum ML [MaxML] and V2), mid-stance (V3), and push-off (minimum AP [MinAP]).^14^

### Statistical analysis

A 2 × 2 × 2 (direction × week × test type) with repeated measures analysis of variance (ANOVA) was performed on each of the seven acceleration measures. An alpha level of 0.05 was used to determine a statistical significance. Seven-item Cronbach's *α* analysis was done to determine the consistency of the gait assessments across the direction of the walk and the two testing sessions for each test type. Cronbach's *α* >0.6 showed at least a moderate level of measurement consistency. All analysis was done using SAS statistical software version 9.4 (SAS Institute, Cary, NC).

## Results

**FIG. 1.**
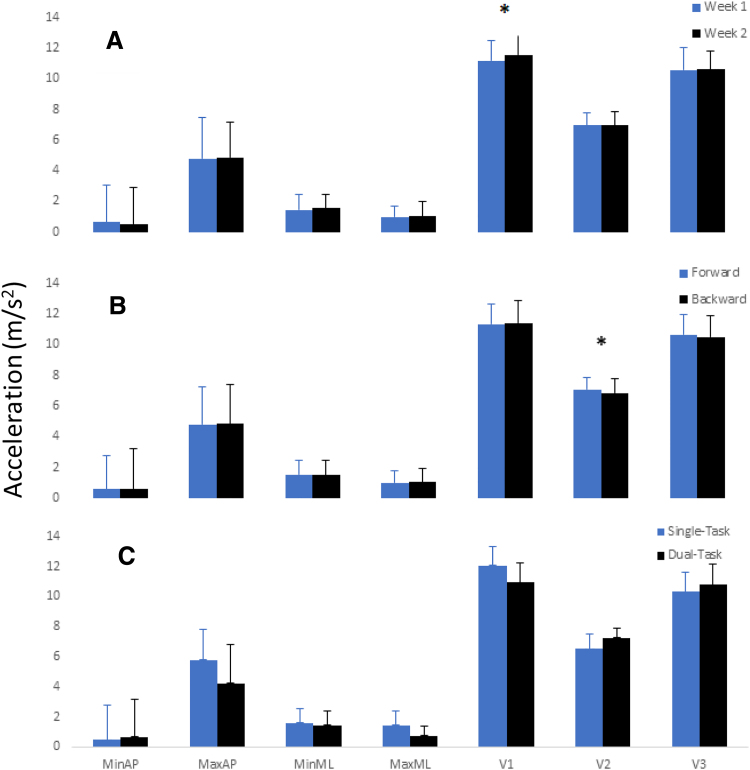
Maximum and minimum AP, ML, and V accelerations presented by **(A)** week, **(B)** direction, and **(C)** task. Significant main effects were observed for V1 and V2 when analyzed by week and direction, respectively. *p < 0.05. AP, anterior-posterior; ML, medial-lateral; V, vertical.

Significant main effects for week and walking direction were found for V1 [F (1,16) = 5.81, *p* = 0.0283], and V2 [*F*(1,16) = 5.11, *p* = 0.0381] with V1 being increased in week 1 versus 2 and V2 being increased on the return to start versus the walk away (Fig. 1). No other differences in the seven measures were observed.

The Cronbach's alpha value for MinML, MaxML, V1, V2, and V3 showed an alpha between 0.60 and 0.73, which shows reliability for those metrics when ST and DT were combined (Table 1). The Cronbach *α* values for MinAP and MaxAP were 0.48 and 0.49, respectively, which indicates less reliability. When done independently, MinML (*α* = 0.62) and MaxML (*α* = 0.68) scored reasonably reliable for the single task. For the dual task, all the metrics scored an alpha level of 0.66–0.80 for the dual task group, whereas the MinAP and MaxAP scored 0.59 and 0.56, respectively.

**Table 1.** Cronbach's ***α*** Values for Single-Task, Dual-Task, and Overall for Each of the Peaks Occurring Within the Medial-Lateral, Anterior-Posterior, and Vertical Acceleration Data

	Cronbach's α		
	ST	DT	Overall
MinAP	0.322635	0.595793	0.487594
MaxAP	0.437762	0.568440	0.490428
MinML	0.624424	0.720071	0.672996
MaxML	0.684305	0.802966	0.739928
V1	0.598379	0.692415	0.670309
V2	0.541100	0.669224	0.608143
V3	0.525893	0.700634	0.608143

AP, anterior-posterior; DT, dual task; MaxAP, maximum AP; MaxML, maximum ML; MinAP, minimum AP; MinML, minimum ML; ML, medial-lateral; ST, single task; V, vertical.

## Discussion

This study suggests that the accelerometers in iPhones are reliable for recording gait data in a nonlaboratory or clinical settings and with trained personnel offering virtual supervision. Our Cronbach's alpha values suggest that our chosen measures could be repeatedly collected with little difference in time (from week to week) or direction (away from the start compared with the return). The introduction of a disruptive mental task appeared to have no effect on the repeatability of these measures suggesting that long distance offsite monitoring may be possible in populations with minor gait disruptions. Findings from this study are consistent with a previous study by Pitt and Chou. In a controlled laboratory environment, they reported high internal consistency as well as high inter-rater reliability for measures of gait nearly identical to the ones in this study. The findings of our study build on the previous work by showing that data can be reliably collected over long distances with video instruction and supervision.

Within this study, we analyzed seven metrics. Those in the ML and vertical axis deemed to have higher Cronbach's alpha compared with the AP axis. This could mean that smartphone technology may be better suited to analyze ML balance or vertical loading but may need to be used more cautiously when it comes to AP gait event detection where pushing and resistance forces are experiences simultaneously during the dual stance phase.^14^ This may be especially true when the phone is held at the waist as it was in this study because others have observed different gait characteristics depending upon instrument placement on the lower trunk.^15^

One limitation of this study was that it was done on healthy young individuals capable of holding the phone at waist height and for whom gait is consistent.^16^ This could not be assumed for clinical populations, especially in populations with balance deficits who may rely on hand movement for stability. Although we tried to introduce gait variability by having a mental test imposed on walking, the mental task had no impact on our measures and was certainly not sufficient to induce significant balance loss. Consequently, clinical populations may prove to be more challenging and future studies should test to see if this method is reliable for different groups.

## Conclusion

Overall, the study shows promising results for using smartphones as tools for the future of telemedicine.

## Abbreviations Used

ANOVA: analysis of variance
AP: anterior-posterior
DT: dual task
IPREP: IUPUI Postbaccalaureate Research Education Program
MaxAP: maximum AP
MaxML: maximum ML
MinAP: minimum AP
MinML: minimum ML
ML: medial-lateral
NIH: National Institutes of Health
ST: single task
V: vertical
